# Supplementary material for: Types, method, and mode of implementation of pain/symptom maps in musculoskeletal pain rehabilitation: A scoping review protocol
Source: PLoS One. 2025 Mar 18;20(3):e0319498. doi: 10.1371/journal.pone.0319498 (PMC11918319; doi:10.1371/journal.pone.0319498)
Supplement: S2 File — (DOCX) [file pone.0319498.s002.docx]

**Appendix II**

**Search strategy framework (Medline)**

1. pain.mp.

2. "pain map*".mp.

3. "pain draw*".mp.

4. "pain chart*".mp.

5. "pain diagram*".mp.

6. "pain app*".mp.

7. 2 or 3 or 4 or 5 or 6

8. "bod* map*".mp.

9. "bod* diagram*".mp.

10. "bod* chart*".mp.

11. "symptom* chart*".mp.

12. "symptom* map*".mp.

13. (manikin* or mannequin*).mp.

14. 8 or 9 or 10 or 11 or 12 or 13

15. 1 and 14

16. 7 or 15

|  |  |
| --- | --- |
