# Supplementary material for: Types, method, and mode of implementation of pain/symptom maps in musculoskeletal pain rehabilitation: A scoping review protocol
Source: PLoS One. 2025 Mar 18;20(3):e0319498. doi: 10.1371/journal.pone.0319498 (PMC11918319; doi:10.1371/journal.pone.0319498)
Supplement: S3 File — (DOCX) [file pone.0319498.s003.docx]

**Appendix III**

**DATA EXTRACTION FORM**

| **Author &**  **Publication Date** | **Purpose of study** | **Study design** | **Participant’s**  **Characteristics** | **Classification by Typical Features** | **Mode of Implementation** | **Mapping Method** | **Nomenclature** | **Pain**  **Measure** | **Note** |
| --- | --- | --- | --- | --- | --- | --- | --- | --- | --- |
|  |  |  | Pain condition: x, n[%]  y, n[%]  z, n[%]  Sex: M/F n[%]:  Age, mean(SD): a(b) | 1 Sca:  2 Reg:  3 dim:  4 Seg:  5 Sex: |  |  |  | Axis I:  Axis II: |  |
|  |  |  |  |  |  |  |  |  |  |
|  |  |  |  |  |  |  |  |  |  |
|  |  |  |  |  |  |  |  |  |  |
|  |  |  |  |  |  |  |  |  |  |
|  |  |  |  |  |  |  |  |  |  |
|  |  |  |  |  |  |  |  |  |  |
|  |  |  |  |  |  |  |  |  |  |
|  |  |  |  |  |  |  |  |  |  |
|  |  |  |  |  |  |  |  |  |  |
|  |  |  |  |  |  |  |  |  |  |
|  |  |  |  |  |  |  |  |  |  |
|  |  |  |  |  |  |  |  |  |  |
|  |  |  |  |  |  |  |  |  |  |
|  |  |  |  |  |  |  |  |  |  |
|  |  |  |  |  |  |  |  |  |  |
|  |  |  |  |  |  |  |  |  |  |
|  |  |  |  |  |  |  |  |  |  |
|  |  |  |  |  |  |  |  |  |  |
|  |  |  |  |  |  |  |  |  |  |
|  |  |  |  |  |  |  |  |  |  |
|  |  |  |  |  |  |  |  |  |  |
|  |  |  |  |  |  |  |  |  |  |
|  |  |  |  |  |  |  |  |  |  |
|  |  |  |  |  |  |  |  |  |  |
|  |  |  |  |  |  |  |  |  |  |
