# Supplementary material for: Types, method, and mode of implementation of pain/symptom maps in musculoskeletal pain rehabilitation: A scoping review protocol
Source: PLoS One. 2025 Mar 18;20(3):e0319498. doi: 10.1371/journal.pone.0319498 (PMC11918319; doi:10.1371/journal.pone.0319498)
Supplement: Attachment — Submitted filename: Response to Reviewers.docx [file pone.0319498.s005.docx]

School of Physical Therapy

Faculty of Health Sciences

Western University

London, Ontario, Canada

January 30, 2025

The Editor-in-Chief

PLOS ONE

**Rebuttal Letter**

***Types, Method, and Mode of Implementation of Pain/Symptom Maps in Musculoskeletal Pain Rehabilitation: A Scoping Review Protocol***

The research team greatly values every comment and suggestion made by the reviewers, and having carefully studied their comments is pleased to respond with possible modifications, additions, or submissions as follows:

***Reviewer #1:*** *Suggestions for Improvement*

*Expand Inclusion Criteria: Consider including studies in other major languages, utilizing translation tools or services to mitigate resource constraints, thereby enriching the diversity of data.*

*Response*:

We recognize that the restriction to English-language publications may mean we exclude a small number of potentially relevant articles. However, we remain confident in this criterion for a number of reasons. These include: 1. the highest-impact journals in the field require at minimum an English-language translation, 2. automatic translation tools like Google translate or newer tools like ChatGPT have yet to demonstrate adequately reliable translation of high-level academic work (e.g., <https://shorturl.at/djq9T>) sometimes making significant errors, and 3. the nature of this review is to identify and characterize common pain maps and scoring strategies, the majority of which will have been used in more than one publication. A one-off pain map published in a single non-English publication is unlikely to have substantive impact on the results of this review, and if it has not been taken up and used by other authors then there is reason to question its utility in the first place. For these reasons, while we acknowledge the potential for excluding a relevant pain map in a non-English paper, we remain confident in the limit to English language publications. We will however acknowledge this as a limitation in the limitation section of the final manuscript.

*Clarify Patient Involvement: Provide more details about how the Patient Partner Advisory Group (PPAG) will be engaged throughout the review process, beyond feedback and suggestions.*

*Response:*
We have added the following lines to the patient partner engagement section: “This protocol has been presented to the PPAG and the group indicated broad support for the importance and impact of the project, while suggesting minor opportunities for improvement (e.g., suggesting areas for deeper investigation or focus). The PPAG will continue to receive periodic updates on the project, after the initial round of interpretation and again prior to submission of the final manuscript. Their feedback will be critical for shaping the focus and messaging from this review.”

*Address Applicability: Expand the discussion on how the findings will be practically applied in clinical and research settings, emphasizing their potential impact on personalized patient care.*

*Response:*

We have added the following paragraph to the Discussion section (Paragraph 3): “As pain mapping evolves from a qualitative tool to a more quantifiable and data-driven resource, it holds considerable promise in both personalized patient care and research outcomes. In clinical settings, pain maps can enhance personalized patient care as a novel communication channel between patient and clinician, as a means of quantifying spatial pain measures to optimize treatment targeting, and enabling clinicians to track progress and evaluate treatment effectiveness beyond the historic reliance on only pain intensity ratings. A more structured approach to pain mapping can complement patient narratives, fostering consistent, patient-centric and evidence-based recommendations. The potential for personalized care is amplified by advancements in artificial intelligence (AI) and machine learning (ML), which when combined with standardized pain maps can detect subtle pain patterns, predict outcomes, and suggest personalized treatment plans (Goldstein et al., 2020). Integrating pain maps into electronic health records (EHRs) enhances the holistic view of a patient's health (Sankaran et al., 2022). From a research perspective, the scoping review will synthesize existing pain mapping methodologies and identify gaps, guiding future studies. Standardizing pain map protocols across studies would enhance comparisons and collaboration, advancing pain management research.”

***Reviewer #2:***

***Abstract***
*Objective: The abstract should clearly specify the review timeframe, key databases, and unique contributions compared to prior studies.*

*Response:*

We have added the following lines to the Abstract: “In preparation for innovative technology-enabled development of quantifiable pain maps, this review will focus on the methodological aspects of recent pain maps. This will include key concepts including scalability, region-specificity, aspect or orientation, segmentation and sex in addition to the reported measurement properties of each mapping approach. This will identify current gaps in knowledge, consistencies in implementation, and inform directions for future development of more person-centric and meaningful pain maps.” and “Key sources of evidence such as Medline, Embase, PsycINFO, CINAHL, Scopus, Web of Science, will be searched from inception to June 5, 2024, including grey literature from reference screening, library and organizational collections such as WorldCat, ProQuest Global Dissertation, Google Scholar, and Google”

*Outcome Clarity: Add distinct primary and secondary outcomes.*

*Response:*

We have also added this line to the abstract: “Primary outcomes include ‘types’ of design: scale, segments, sex, orientation, region; pain mapping methods: marking, shading, checking; and mode of implementation: paper, digital, etc. Secondary outcomes include axis I: pain location, extent or distribution; and axis II: pain severity, intensity, and quality.”

***Introduction*** *Please clearly define the distinction between "methods" and "modes" of implementation to avoid reader confusion.*

*Response:*

We have added this line(s) to the second paragraph of the introduction: “The mapping ‘method’ refers to the specific procedures or techniques used to assess and capture the spatial characteristics of pain (e.g., shading, marking, grid, etc.), while the ‘mode’ of assessment refers to the mediums or platforms through which the patient interacts with the pain mapping tool (e.g., paper and pen, digital, etc.) (Jaatun et al., 2013)”

*Highlight the role of advanced technologies, such as machine learning, more explicitly.*

*Response:*

We have added this paragraph (paragraph 4) to the introduction section: “Machine learning (ML) and artificial intelligence (AI) are transforming pain mapping by enabling more accurate and comprehensive analysis of pain patterns beyond a simple summation of ‘areas selected’ or ‘pixels shaded’. These technologies can process large datasets of pain maps to identify subtle trends in pain location, extent or distribution, intensity, and changes over time, helping to create personalized treatment plans. Goldstein et al., (2020) have shown that ML/AI models have the potential to integrate patient-specific factors like demographics and medical history to reduce trial-and-error in pain management and predict the progression of musculoskeletal conditions. Furthermore, ML can contribute to the development of standardized and objective pain maps, improving consistency and accessibility for diverse populations. Sankaran et al., (2022) argued that the integration of AI/ML with electronic health records (EHRs) promises to streamline data collection, advancing both clinical practice and research in pain management. In essence, ML and AI are driving a paradigm shift in pain mapping by offering more accurate, comprehensive, and personalized insights into pain patterns (Sankaran et al., 2022). These advancements not only improve clinical outcomes but also promise to make pain management more efficient, effective, and accessible across a variety of patient populations.”

***Methods***
*Please Clarify exclusion criteria for artistic body maps and imaging modalities. Examples would be helpful.*

*Response:*

In the method (intervention) section, we replaced the second sentence with this line: “Studies lacking definite patient-reported pain/symptom measurement intervention such as X-ray or CT or MRI scans in pain assessment will be excluded. Additionally, studies using artistic body maps which are life-sized, mostly caricature, images that qualitatively represent life experiences with symbols at corresponding body locations whose meaning is exclusive to the creator (Hulley & Young, 2022), will be excluded.”

*Consider justifying why non-English studies are excluded rather than providing translations or summaries, as this may limit generalizability.*

*Response:*

We have replaced the second, third, and fourth sentences in the method (information source) section with the following sentence: “Considering the broadness of the search scope, the review team anticipates that only a few articles in other languages may be relevant to our study for the following reasons: 1. the highest-impact journals in the field require at minimum an English-language translation, 2. automatic translation tools like Google translate or newer tools like ChatGPT have yet to demonstrate adequately reliable translation of high-level academic work (e.g., <https://shorturl.at/djq9T>) sometimes making significant errors, and 3. the nature of this review is to identify and characterize common pain maps and scoring strategies, the majority of which will have been used in more than one publication”

*Search Strategy:
Include an explicit description of the keywords and Boolean operators used in the search strategy. The framework of the search strategy could benefit from more examples of "grey literature" sources.*

*Response:*

We have replaced the second sentence in the method (search strategy) section with the following line(s): “Using relevant keyword search, “pain” will be the overarching term and/or keyword for variety of musculoskeletal conditions (Coraci et al., 2023), and keywords for pain/symptom mapping interventions will also be applied, as follows: (pain) AND ("pain map*" OR "body map*" OR "pain app*" OR "pain diagram*" OR "body diagram*" OR "pain draw*" OR "pain chart*" OR “bod* chart*” OR "symptom chart*" OR "symptom map*" OR manikin* OR mannequin*).”

***Screening and Selection:***
*Please add more detail on the process of resolving conflicts between reviewers during screening.
Specify whether quality checks (e.g., inter-rater reliability) will be conducted on reviewer decisions.*

*Response:*

We have replaced the method (Selection of Sources of Evidence) section with the following paragraph: “All identified citations will be uploaded to Covidence, and the screening process will be documented in a PRISMA flowchart (Peters et al., 2021), including reasons for exclusion. Duplicate citations will be removed through automatic text screening. Two independent reviewers will screen titles and abstracts to identify relevant studies, followed by full-text screening to assess eligibility. Disagreements will be resolved through discussion between the reviewers; if consensus is not reached, a third experienced reviewer will be consulted to make the final decision. However, the nature of the review occasioned by broadness of the topic, diversity of study designs, less focus on quality assessment, and the potential inconsistencies in framework for deploying intervention, data charting priorities, and iterative and reflective process (Arksey & O’Malley, 2005), complicates defining a kappa threshold and makes it less important to report inter-rater reliability.”

***Data Extraction:***
*Expand on how the data extraction form will evolve during the pilot phase, detailing examples of proposed modifications.*

*Response:*

We have added the following lines to the method (data charting) section: “Modifications might include creating new columns to assess additional details about pain mapping that were unanticipated, potentially including different methods for data analysis (description of how pain mapping data was quantified), views (lateral, dorsal, etc), scoring approach, renaming, and merging columns to adequately present findings.”

***Results and Synthesis***
*Clearly state whether thematic analysis will be incorporated into the synthesis or if results will rely purely on descriptive statistics. The manuscript mentions narrative synthesis but does not outline a clear strategy for integrating findings across diverse study designs.*

*Response:*

To clarify, by ‘narrative synthesis’ we are referring to a broad qualitative interpretation of the current trends and directions in the field rather than alluding to a specific qualitative technique like Narrative Thematic Analysis. We have added these lines to the method (result and synthesis) section: “The synthesis of the results will rely on both descriptive statistics and narrative synthesis. Descriptive statistics will summarize quantitative data, such as the frequency and distribution of different types, methods, and modes of pain mapping. Narrative synthesis will be used to integrate and describe qualitative findings, focusing on trends, patterns, and variations across the studies. This approach will allow for a comprehensive understanding of the literature, identifying key insights as well as inconsistencies or gaps in the research.”

***Discussion***
*The authors rightly discuss the limitations of resource constraints. However, elaborating on strategies to mitigate these, such as collaboration with translators, would be beneficial.*

*Response:*

We have replaced the second and third sentences in the second paragraph in the discussion section with this sentence “However, in keeping with the broad scope of this review, there is a limitation in language inclusivity. This review focuses on identifying the characteristic features of common pain maps and scoring strategies, which are typically reported in multiple publications, although the highest-impact journals in the field require, at a minimum, an English-language translation.”

***Future Directions:***
*Provide concrete recommendations for future research based on the potential findings.*

*Response:*

We have added this section (future direction) to the manuscript: “This review will describe and synthesize recent and current trends, uses, modalities, and gaps in the use of pain mapping for research or clinical use. This is a critical step towards the development of more meaningful and informative maps that leverage new and emerging technologies while centering patient values and needs related to communicating pain. Next steps are expected to include presentation of results to the PPAG, linkages with experts in the field of machine learning, and rigorous design and testing of novel approaches to pain mapping.”

***References***
*The references are comprehensive but verify that they adhere to the journal’s citation format, particularly for online resources.*

*Response:*

All references were updated to the journal’s format and crosschecked to confirm that the list is complete, and none have been retracted. Two references, Eliav, E., & Gracely, R. (2008) and Turk *n.d* were modified. In addition, four new references were added to the manuscript.

1. Goldstein, P., Ashar, Y., Tesarz, J., Kazgan, M., Cetin, B., & Wager, T. D. (2020). Emerging Clinical Technology: Application of Machine Learning to Chronic Pain Assessments Based on Emotional Body Maps. *Neurotherapeutics: The Journal of the American Society for Experimental NeuroTherapeutics*, *17*(3), 774–783. https://doi.org/10.1007/s13311-020-00886-7
2. Hulley, S., & Young, T. (2022). Body Map Storytelling: Exploring identity with lifers convicted of murder using the doctrine of ‘joint enterprise.’ *Prison Service Journal*, *2022*(261), Article 261.
3. Jaatun, E. A. A., Haugen, D. F., Dahl, Y., & Kofod-Petersen, A. (2013). Proceed with Caution: Transition from Paper to Computerized Pain Body Maps. *Procedia Computer Science*, *21*, 398–406. https://doi.org/10.1016/j.procs.2013.09.052
4. Sankaran, R., Kumar, A., & Parasuram, H. (2022). Role of Artificial Intelligence and Machine Learning in the prediction of the pain: A scoping systematic review. *Proceedings of the Institution of Mechanical Engineers, Part H: Journal of Engineering in Medicine*, *236*(10), 1478–1491. https://doi.org/10.1177/09544119221122012

We thank the reviewers for their time in reviewing our manuscript and believe it has become stronger as a result of this process. We look forward to the results of the next round of review.

Sincerely,

Ukponaye D. Eboigbe

Corresponding author

ueboigbe@uwo.ca
